# Reproducibility of native myocardial T1 mapping in the assessment of Fabry disease and its role in early detection of cardiac involvement by cardiovascular magnetic resonance

**DOI:** 10.1186/s12968-014-0099-4

**Published:** 2014-12-05

**Authors:** Silvia Pica, Daniel M Sado, Viviana Maestrini, Marianna Fontana, Steven K White, Thomas Treibel, Gabriella Captur, Sarah Anderson, Stefan K Piechnik, Matthew D Robson, Robin H Lachmann, Elaine Murphy, Atul Mehta, Derralyn Hughes, Peter Kellman, Perry M Elliott, Anna S Herrey, James C Moon

**Affiliations:** The Heart Hospital, 16-18 Westmoreland Street, London, W1G 8PH UK; Department of Cardiology, IRCCS Policlinico San Matteo Hospital, Pavia, Italy; Institute of Cardiovascular Science, University College London, London, WC1E 6BT UK; Department of Cardiovascular, Respiratory, Nephrologic, Anaesthesiologic and Geriatric Science, La Sapienza University of Rome, Rome, Italy; Oxford Centre for Clinical Magnetic Resonance Research, Department of Cardiovascular Medicine, University of Oxford, Oxford, OX3 9DU United Kingdom; The National Hospital for Neurology and Neurosurgery, Queens Square, London, UK; Lysosomal Storage Disorders Unit, Royal Free Hospital and University College London, London, UK; National Heart, Lung, and Blood Institute, National Institutes of Health, Bethesda, MD USA

**Keywords:** Cardiovascular magnetic resonance, T1 mapping, Speckle-tracking strain, Diastolic function, Fabry disease

## Abstract

**Background:**

Cardiovascular magnetic resonance (CMR) derived native myocardial T1 is decreased in patients with Fabry disease even before left ventricular hypertrophy (LVH) occurs and may be the first non-invasive measure of myocyte sphingolipid storage. The relationship of native T1 lowering prior to hypertrophy and other candidate early phenotype markers are unknown. Furthermore, the reproducibility of T1 mapping has never been assessed in Fabry disease.

**Methods:**

Sixty-three patients, 34 (54%) female, mean age 48 ± 15 years with confirmed (genotyped) Fabry disease underwent CMR, ECG and echocardiographic assessment. LVH was absent in 25 (40%) patients. Native T1 mapping was performed with both Modified Look-Locker Inversion recovery (MOLLI) sequences and a shortened version (ShMOLLI) at 1.5 Tesla. Twenty-one patients underwent a second scan within 24 hours to assess inter-study reproducibility. Results were compared with 63 healthy age and gender-matched volunteers.

**Results:**

Mean native T1 in Fabry disease (LVH positive), (LVH negative) and healthy volunteers was 853 ± 50 ms, 904 ± 46 ms and 968 ± 32 ms (for all p < 0.0001) by ShMOLLI sequences. Native T1 showed high inter-study, intra-observer and inter-observer agreement with intra-class correlation coefficients (ICC) of 0.99, 0.98, 0.97 (ShMOLLI) and 0.98, 0.98, 0.98 (MOLLI). In Fabry disease LVH negative individuals, low native T1 was associated with reduced echocardiographic-based global longitudinal speckle tracking strain (−18 ± 2% vs −22 ± 2%, p = 0.001) and early diastolic function impairment (E/E’ = 7 [6–8] vs 5 [5–6], p = 0.028).

**Conclusion:**

Native T1 mapping in Fabry disease is a reproducible technique. T1 reduction prior to the onset of LVH is associated with early diastolic and systolic changes measured by echocardiography.

## Background

Fabry disease is a rare but treatable X-linked disorder of lysosomal metabolism caused by reduced or absent activity of the alpha galactosidase enzyme, resulting in lysosomal sphingolipid accumulation in a number of different organs. Since the advent of renal replacement therapy, the principle driver on mortality in Fabry disease is cardiac. Left ventricular hypertrophy (LVH), valve thickening, myocardial scarring, progression to heart failure and sudden arrhythmic death may occur [1,2]. Fabry disease patients can be treated with enzyme replacement therapy (ERT) [3]. A number of major challenges in the clinical care of patients with potential Fabry disease remain, such as: (i) the differential diagnosis of Fabry disease-related LVH vs other causes of LVH; (ii) the detection of early cardiac involvement in genetically diagnosed Fabry disease patients (particularly in female heterozygotes); (iii) Timing of commencement and monitoring of ERT. The biology of myocardial storage (hypertrophy, fibrosis, cell loss) is not understood, but there is suggestion that therapy should be started early before changes are permanent [4]. However, treatment is sufficiently expensive that early therapy would have societal cost implications [5-7]. New, non-invasive markers of myocardial storage are therefore needed.

Recent advances in cardiovascular magnetic resonance (CMR) allow measurement of native myocardial T1 using a single, short breath-hold mapping sequence [8]. Native T1 is known to be higher in fibrosis, edema and amyloid, and lower in iron overload and focal fat infiltration [9-11]. In Fabry disease with LVH, both our group and another have shown that native myocardial T1 is substantially lower compared to other LVH aetiologies [12,13] and around half of Fabry disease patients without LVH have T1 lowering when compared to healthy volunteers [13]. The known lowering of T1 by fat and the early spectroscopic results suggest that native T1 may be directly measuring myocardial storage in Fabry disease [12]. Native T1 mapping therefore has the potential role in clinical evaluation (prognosis, differential diagnosis and early detection) and management (timing of therapy and follow up) of Fabry disease patients and as a tool to help in understanding the biology of myocardial storage. However, several questions regarding the use of T1 mapping in Fabry disease remain, particularly its reproducibility and whether low native T1 in LVH negative patients is a real marker of early disease with potential functional consequences.

In this study we investigated the reproducibility of native T1 assessment in Fabry disease and the relationship of native T1 in Fabry disease patients with no LVH, looking for functional (electrocardiographic, mechanical) correlations of T1 reduction.

## Methods

This research received approval from the local research ethics committee and all participants provided written informed consent. Patients were prospectively recruited from the department of inherited cardiovascular disease at The Heart Hospital, London, UK. All patients had a genetically confirmed diagnosis of Fabry disease. This population was compared with gender and age matched cohorts of healthy volunteers: one for the whole population of Fabry disease, one for the male cohort and one for the female cohort, selected from a pool of 63 healthy volunteers, as previously described [13].

CMR was performed on a 1.5 T magnet (Avanto, Siemens Medical Solutions). Left ventricular volume, mass and ejection fraction were calculated from steady state free precession cine imaging using a thresholding method. LVH was defined as an elevated indexed left ventricular mass based on body surface area (BSA) normalized cut-off values and stratified for age and gender (Table 1) [14].

**Table 1:** **BSA-normalized LVmass cut-offs age and gender stratified for LV-hypertrophy definition**

	**20–29 years**	**30–39 years**	**40–49 years**	**50–59 years**	**60–69 years**	**70–79 years**
Females LVmass/BSA (g/m2)	62(47–77)	62(47–77)	63(48–77)	63(48–78)	63(48–78)	63(49–78)
Males LVmass/BSA (g/m2)	76(59–93)	75(59–92)	75(58–91)	74(57–91)	73(57–90)	73(56–89)

Adapted from Maceira et. al.

BSA: body surface area; LV: left ventricle.

Native T1 measurement was performed using a Shortened Modified Look Locker Inversion recovery (ShMOLLI) sequence. In the reproducibility group a second sequence, Modified Look Locker Inversion recovery (MOLLI – sampling scheme 5 s(3 s)3 s with motion correction) was also performed and the patient removed from the scanner, to be re-scanned later in the day; in this group, ShMOLLI and MOLLI T1 assessment were then repeated. The minimum and maximum time between scan 1 and 2 was 8 to 40 minutes. A shim box was placed tightly over the heart as recommended [15]. Optimal gating and breath-holding were ensured and raw images and error maps were examined for potential image artefacts during scanning, to allow an immediate repeat of suboptimal measurements [8]. Following the second T1 assessment, standard late gadolinium enhancement (LGE) imaging was performed on all patients. In patients not undergoing reproducibility, LGE was performed at the end of the first and only scan. Both ShMOLLI and MOLLI T1 sequences were performed using previously published algorithms [16,17]. For ShMOLLI, the resulting pixel-by-pixel color T1 maps were displayed using a customised 12 bit look up table where normal myocardium was green, increasing T1 red and decreasing T1 blue. The color-map was immediately available after data acquisition. A region of interest (ROI) was drawn directly on each T1 map using ARGUS software (Siemens, Erlangen, Germany) and T1 values averaged between all pixels. T1 was measured in the basal to mid septum in the short axis plane taking care to avoid the blood/myocardial boundary. A second ROI was drawn in basal and mid infero-lateral wall and later compared with LGE images. For test-retest reproducibility study, this process was done for both ShMOLLI and MOLLI sequences.

The analyses were carried out by two blinded observers (SP and DS): SP analyzed the same ShMOLLI and MOLLI mid short axis picture twice for intra-observer reproducibility, DS analysed all studies (including re-test) once, making intra-observer, inter-observer and inter-study data available. For these analyses, all scans were anonymized, i.e. scan 1 and 2 split up.

All patients enrolled underwent ECG and echocardiographic examination within two days of the CMR study. ECG analysis comprised the assessment of cardiac rhythm and axis, PR interval, QRS interval, indices of LVH including RE score [18]. Echocardiography was performed using commercially available systems (Vivid 7 or 9, GE Healthcare). A routine study was performed, according to standard protocols of the European Society of Cardiology, including evaluation of diastolic function with conventional Doppler based measurements (trans-mitral E/A ratio, E wave deceleration time, pattern of pulmonary vein flow); Tissue Doppler was performed to assess S’ and E’ mitral annular velocities at the septal and lateral corners, with the measurements being averaged. The E/E’ ratio was derived as a measure of LV filling pressures. Digital routine grey scale two-dimensional cine loops from three consecutive beats were obtained from standard apical and short axis views. Systolic 2D strain was calculated by real-time tracking of natural acoustic markers during consecutive frames by 2D strain software as previously described [19]. All data was prospectively and blindly analysed for global and infero-lateral longitudinal, circumferential and radial strain.

Exclusion criteria for this study were usual contraindications to perform a CMR scan. Patients with eGFR <30 ml/min were not administered gadolinium contrast and patients with sub-optimal acoustic window or frame rates were excluded from the speckle tracking strain analysis.

### Data analysis and statistics

Data were analysed with JMP® 8.0.2 software. Data following a parametric distribution (Shapiro Wilk test p > 0.05) was described using mean ± standard deviation. Non-parametric data was described using median and interquartile range. Count and percent were used to describe categorical data. The difference between groups was compared with either the Student t test (parametric) or the Mann Whitney U test (non parametric) and Fisher’s exact test for categorical data Correlations between parameters are described either with Pearson R or with Spearman ρ (rho), depending on their distribution. Inter-study, intra-observer and inter-observer reproducibility of native T1 measurements was assessed by calculating the Intraclass Correlation Coefficient (ICC) with Pearson correlation and Bland Altman plots and the coefficient of variance (COV). A p value <0.05 was considered statistically significant.

## Results

### Who is in study: baseline characteristics

Sixty-three patients were included in the study. Of the 63 patients, 44 had previously been studied from a cohort previously published [13]. Two of these were rescanned with reproducibility and 19 new patients were added. The present study added ECG and echocardiographic data for clinical correlations.

Baseline characteristics of Fabry disease and healthy volunteers are showed in Table 2. Seven patients were not administered gadolinium contrast, either due to severe renal impairment (estimated glomerular filtration rate, eGFR, <30 ml/min, n = 5), or patient refusal (n = 2). Twelve patients with sub-optimal acoustic window or frame rates were excluded from speckle tracking strain analysis.

**Table 2:** **Baseline characteristics of the study population**

**Parameter**	**FD global cohort (n = 63)**	**FD LVH negative (n = 25)**	**FD LVH positive (n = 38)**	**Healthy volunteers (n = 63)**
Age (years)	48 ± 15	39 ± 16	54 ± 11	47 ± 16
Sex (M/F)	29/34	6/19	23/15	29/34
BSA (m2)	1.8[1.7, 2]	1.7[1.6,1.8]	1.9[1.7, 2]	1.9[1.7, 2]
LVH (%)	38(60)	0	38(100)	0
ERT (%)	49(78)	14(56)	35(92)	0
Any LGE (%)	30(54)	4(17)	26(79)	0
LGE infero-lateral wall	29(51)	4(17)	25(76)	0

Data are expressed as n(percentage) or mean ± standard deviation.

BSA: body surface area; LVH: left ventricular hypertrophy; ERT: enzymatic replacing therapy; LGE: late gadolinium enhancement.

### Age and gender reference ranges for T1

Native T1 is known to be influenced by gender and (to a much lesser extent) age [12,20]. For comparison with healthy controls therefore, on the same scanner/set-up, normal ShMOLLI ranges (and therefore limits of normal) were constructed for Fabry disease male and female cohorts. Age and gender matched comparator groups selected from a pool of 63 healthy volunteer scans were used to identify what the normal and low native T1 is for male and female population. Using these, the native T1 ranges were (mean, SD, lower limit of normal): mean 968 ± 32 ms, lower limit 904 ms in total population; mean 956 ± 27 ms, lower limit 902 ms in male subgroup and mean 978 ± 34 ms, lower limit 910 ms in female subgroup.

Mean septal T1 in Fabry disease males (n = 29) was lower than in females (n = 34), (841 ± 46 ms vs 901 ± 45 ms, p <0,0001).

**Figure 1:**
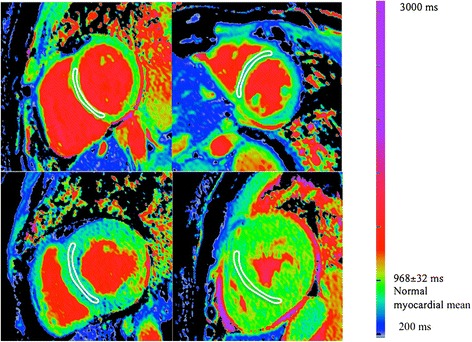
**Native T1 mapping in Fabry disease using ShMOLLI at 1.5 T.** Top left: normal. Top right: a Fabry disease subject without LVH but clear myocardial T1 reduction – the myocardium is blue. Bottom left: Typical T1 when LVH present: the myocardial T1 is lower than without LVH and the basal infero-lateral wall has T1 elevation with a normal (pseudonormal?) surrounding area. Bottom right: rarely (4 patients), Fabry disease has a normal T1.

CMR characteristics of Fabry disease individuals divided into two groups (based on the presence or absence of LVH) are shown in Table 3. ECG and Echocardiographic characteristics of Fabry disease subjects are shown in Table 4. The QRS width was directly correlated with left ventricular mass, Pearson R = 0.72, p < 0.0001. An example of T1 mapping in Fabry disease individuals is in Figure 1.

**Table 3:** **CMR characteristics of Fabry disease patients divided into LVH positive/LVH negative and Healthy volunteers**

	**Healthy volunteers (n = 63)**	**FD LVH negative (n = 25)**	**FD LVH positive (n = 38)**	**p-value** ^**†**^
LV Mass i (g/m2)	66 ± 14	72 ± 13	132 ± 46	<0.01
Max wall thickness (mm)		8 ± 2	16 ± 4	<0.01
LVEDVi (ml/m2)	72 ± 13	70 ± 15	67 ± 16	0.30
LVESVi (ml/m2)	24 ± 7	17 ± 6	16 ± 6	0.10
LVEF (%)	67 ± 5	74 ± 6	78 ± 7	0.05
Left atrial area i (cm2/m2)	11 ± 2	12 ± 3	13 ± 3	0.03
LGE	0	4(17)	26(79)	<0.01
infero-lateral wall		4(17)	25(76)	<0.01
extensive		0	2(6)	
RV inserction points		0	1(3)	
Average septal T1 (ms)*	968 ± 32	904 ± 46	853 ± 50	<0.01
Average infero-lateral T1 (ms)*		894 ± 65	903 ± 45	0.27
Average infero-lateral T1 (ms)*				
LGE yes		954 ± 15	919 ± 63	0.01
LGE no		888 ± 41	848 ± 32	<0.01

Data are expressed as n (percentage) or mean ± standard deviation.

*native T1 measured by ShMOLLI sequences.

^†^p value between LVH negative and LVH positive groups.

LVEDV: left ventricular end-diastolic volume; LVESV: left ventricular end-systolic volume; LGE: late gadolinium enhancement; LVEF: left ventricular ejection fraction, RV: right ventricle.

**Table 4:** **ECG and Echocardiographic characteristics of Fabry disease patients divided into LVH negative and LVH positive**

	**LVH negative (n = 25)**	**LVH positive (n = 38)**	**p-value**
PR interval (ms)	149 ± 32	158 ± 29	0.25
QRS interval (ms)	92 [85, 100]	108 [96, 130]	<0.01
Axis (degree)	60 [47, 74]	38 [−12, 63]	<0.01
TWI	3 (12)	30(79)	<0.01
Sokolow (Sv1 + RV5/6) (mm)	32 ± 9	37 ± 18	0.06
RE score ≥4	3 (12)	32 (84)	<0.01
E/E’	6 [5,8]	10 [8,13]	<0.01
Systolic velocity Doppler TDI (m/s)	0.09 ± 0.02	0.06 ± 0.01	<0.01
Left atrial volume (ml)	60 ± 18	91 ± 28	<0.01
Global longitudinal strain (%)	−20 ± 3	−13 ± 4	<0.01
Infero-lateral longitudinal strain (%)	−21 ± 3	−13 ± 4	<0.01
Global basal radial strain (%)	38 ± 19	34 ± 12	0.22
Infero-lateral basal radial strain (%)	44 ± 14	34 ± 15	0.02
Global basal circumferential strain (%)	−18 ± 4	−15 ± 5	0.03
Infero-lateral basal circumf strain (%)	−13 ± 5	−10 ± 8	0.05
TAPSE (mm)	23 ± 4	20 ± 3	<0.01

Data are expressed as n (percentage) or mean ± standard deviation or median [interquartile range].

TWI: T wave inversion; TDI: Tissue Doppler Imaging; TAPSE: tricuspid annular plane systolic excursion.

**Figure 2:**
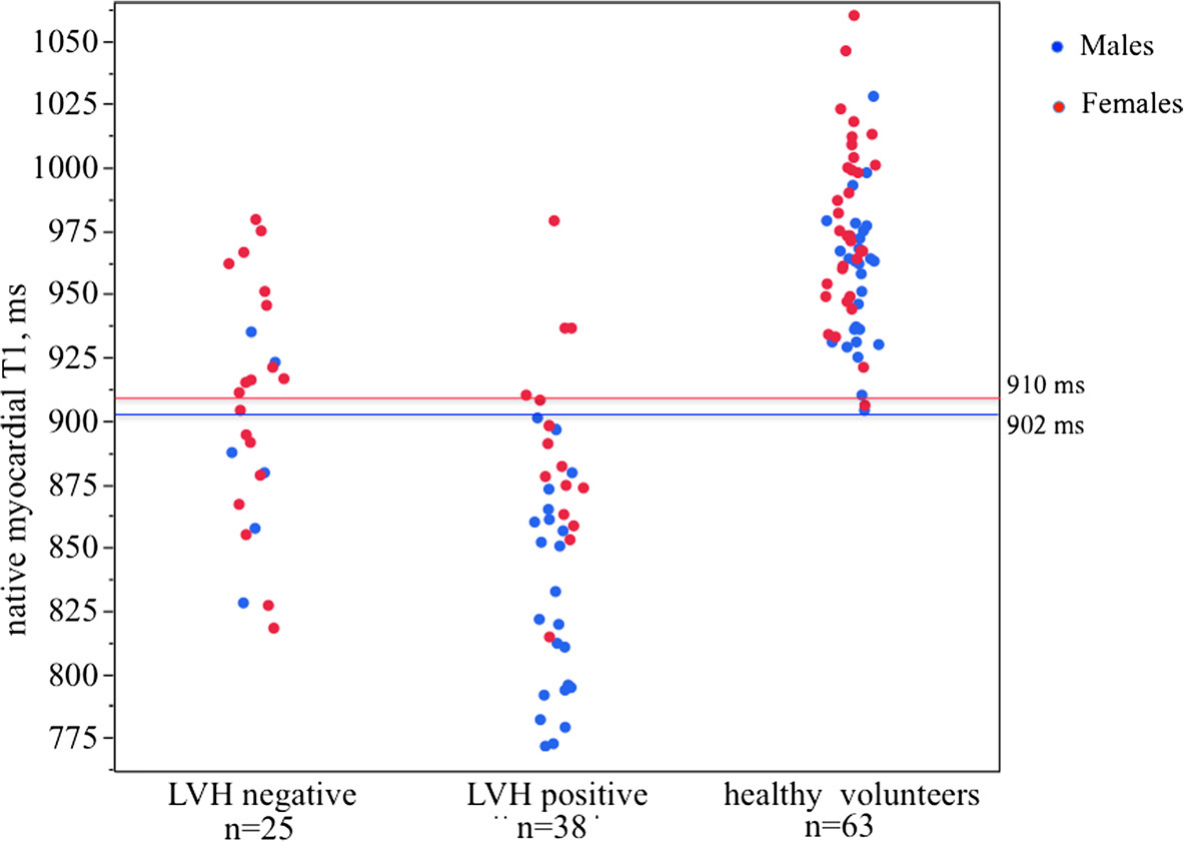
**Mean septal T1 in LVH negative patients (n = 25), LVH positive patients (n = 38) and healthy volunteers (n = 63).** Red solid line indicates -2SD below the mean native T1 of females healthy volunteers. Blue solid line indicates -2SD below the mean native T1 of males healthy volunteers.

### T1 in Fabry disease: LVH positive vs LVH negative

Septal T1 in Fabry disease subjects LVH positive (n = 38) was lower than in LVH negative (n = 25) and healthy volunteers (n = 63), (853 ± 50 ms vs 904 ± 46 ms vs 968 ± 32 ms respectively, p < 0.0001) (Figure 2); there was a weak negative correlation between native T1 and hypertrophy (expressed as left ventricular mass), Pearson R = −0.26, p = 0.03. Of the LVH positive Fabry disease subjects, 34 (89%) had low T1 (<902 ms for males, <910 ms for females). Of the Fabry disease LVH negative subjects, 12 (48%) had low T1.

**Figure 3:**
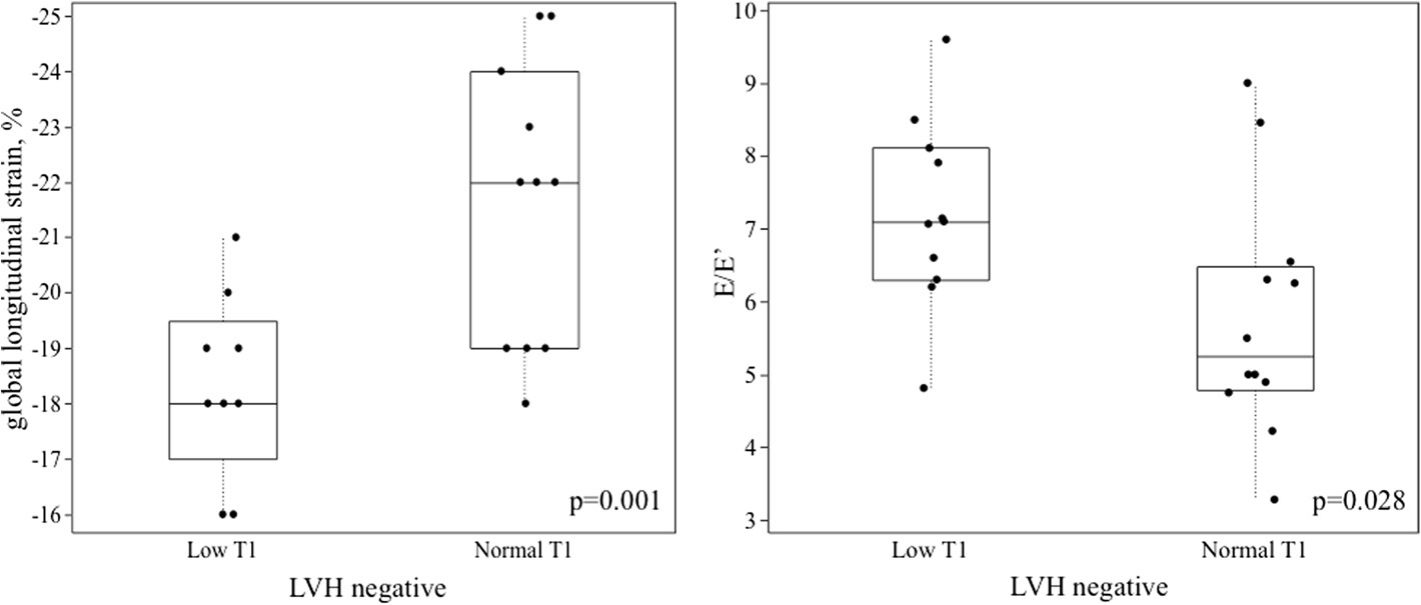
**Systolic and diastolic function in LVH negative Fabry disease patients.** Global longitudinal speckle tracking strain of LVH negative-T1↓ subjects *vs* LVH negative-T1*N* subjects (left); E/E’ of LVH negative-T1↓ subjects *vs* LVH negative-T1*N* subjects (right).

### T1 in LVH positive population

Thirty-four of thirty-eight patients (89%) of LVH positive patient had low septal T1, ranging from 771 ms to 908 ms. The 4 Fabry disease subjects with ‘normal’ septal T1 were females, 3 of them with mild LVH or apical prevalent LVH and 2 of them with basal infero-lateral LGE and >3 years of ERT (minimum 5, maximum 12 years). One subject, 44 years, had severe concentric hypertrophy, T1 = 978 ms, 2 years of ERT and extensive LGE. For this small group (n = 4), standard and advanced echo and CMR parameters were the same as the low T1 LVH positive group. The ECG (QRS) was narrower in this group however (94 ± 7 ms vs 117 ± 23 ms, p < 0.01).

Regionally, native T1 in the infero-lateral wall tracked the presence (n = 25) or absence (n = 9) of LGE in this area (LGE+ native T1 919 ± 63 ms vs LGE- native T1 848 ± 32 ms, p < 0.01).

### T1 in LVH negative population

Of the LVH negative subjects who were administered contrast (n = 23), 4 (17%) had LGE, always in the infero-lateral wall. This was associated with normal mean T1 in the infero-lateral segment as compared with lower T1 in subjects without LGE (954 ± 15 ms vs 888 ± 41 ms, p < 0.0001). We found no correlation between mean septal T1 and LGE. Mean septal T1 was comparable in patients with and without LGE (902 ± 49 ms vs 908 ± 46 ms, p 0.8)

Fabry disease patients without LVH had a mean septal T1 at the lower limit of normal range (904 ± 46 ms), ranging from 818 ms to 979 ms (Figure 2).

Based on the ShMOLLI mean value of 956 ± 27 ms for males and 978 ± 34 ms for females, we divided our population into four groups, according to presence/absence of LVH and low/normal native septal T1, (−2SD below healthy mean: 902 ms cut-point for males and 910 ms cut-point for females). Group 1: LVH negative-T1*N*, n = 13; Group 2: LVH negative-T1↓, n = 12; Group 3: LVH positive-T1↓, n = 34; Group 4: LVH positive-T1*N*, n = 4.

### Significance of T1 lowering in LVH negative Fabry disease subjects.

There were 25 Fabry disease patients without LVH: 13 with normal T1 and 12 with low T1. Comparing these groups, the ECG was the same between groups. Standard CMR and Echo parameters for heart size and function were the same, but advanced echocardiography techniques revealed differences in systolic and diastolic function with slightly lower global longitudinal speckle tracking strain, and reduced diastolic function based on E/E’ in LVH negative-T1↓ subjects (Figure 5). This also tracked the degree of T1 lowering (T1 vs strain Pearson R −0.41, p 0.07; T1 vs E/E’ Spearman rho = −0.51, p = 0.01), Table 5 and Figure 3. Supporting diastolic impairment, left atrial size was bigger in LVH negative-T1↓ subjects than in LVH negative-T1*N*.

**Table 5:** **Echocardiographic, CMR, ECG characteristics of LVH negative individuals with normal (LVH negative-T1** ***N*** **) or low T1 (LVH negative-T1↓)**

	**LVH negative-T1** ***N*** **, (n = 13)**	**LVH negative-T1↓, (n = 12)**	**p-value**
E/E’	5[5,6]	7[6,8]	0.03
Global longitudinal strain (%)	−22 ± 2	−18 ± 2	<0.01
Infero-lateral longitudinal strain (%)	−23 ± 3	−20 ± 4	0.01
Global basal radial strain (%)	37 ± 24	39 ± 13	0.36
Infero-lateral basal radial strain (%)	45 ± 15	42 ± 15	0.70
Global basal circumferential strain (%)	−18 ± 5	−18 ± 4	0.99
Infero-lateral basal circumf strain (%)	−15 ± 6	−12 ± 3	0.12
TAPSE (mm)	23 ± 4	23 ± 4	0.96
Systolic velocity Doppler TDI (m/s)	0.09 ± 0.02	0.08 ± 0.02	0.36
PR interval (ms)	148 ± 22	150 ± 42	0.45
QRS interval (ms)	93 ± 9	95 ± 11	0.26
TWI	1(8)	2(17)	0.56
RE score ≥4	2(15)	1(8)	0.46
Left atrial area i (cm2/m2)*	11 ± 2	13 ± 2	<0.01
LVEDVi (ml/m2)*	67 ± 7	72 ± 19	0.22
LV mass i (g/m2)*	69 ± 10	76 ± 16	0.19

Data are expressed as n(percentage) or mean ± standard deviation or median [interquartile range].

TDI: Tissue Doppler Imaging; TAPSE: tricuspid annular plane systolic excursion; TWI: T wave inversion; LVEDV: left ventricular end-diastolic volume;

*by CMR.

**Figure 4:**
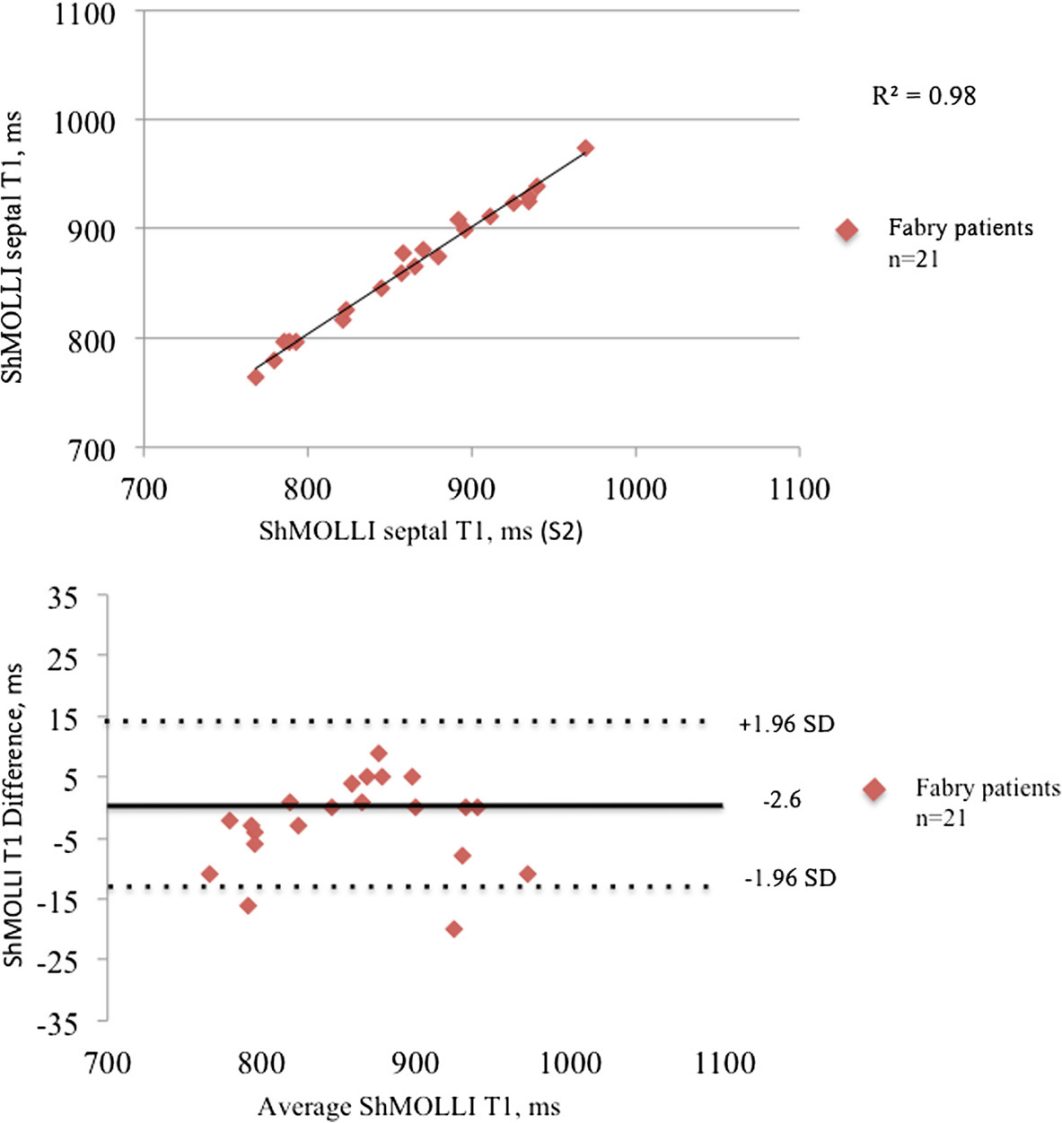
**Reproducibility of ShMOLLI sequences.** ShMOLLI inter-study correlation (upper pannel) and Bland Altman analysis (lower pannel).

Considering the cut off value of 902 ms for males and 910 ms for females, T1 had 48% sensitivity (i.e. half did not have low T1), but 99% specificity in distinguishing between Fabry disease LVH negative subjects and healthy volunteers (Receiver operating characteristic AUC 0.73).

ShMOLLI and MOLLI sequences derived exactly the same results but with, as previously described, a sequence design related 60 ms offset (MOLLI higher) [21].

### Reproducibilty

T1 mapping was highly reproducible regardless of methodology used (Table 6): inter-study reproducibility for ShMOLLI sequences showed an intraclass correlation coefficients (ICC) of 0.992 and relative difference between the means of −0.3% (95%CI −4.6% to 4.0%) (Figure 4). Intra-observer and inter-observer reproducibility were similarly very good with relative difference between the means of −0.3% (95% CI −4.6% to 4.0%) and −0.8% (95% CI −3.5% to 5.0%) respectively. ShMOLLI and MOLLI sequences showed the same reproducibility.

**Table 6:** **ShMOLLI and MOLLI T1 reproducibility**

	**Inter-study ICC (COV%)**	**Intra-observer ICC (COV%)**	**Inter-observer ICC (COV%)**
ShMOLLI T1	0.992(0.8%)	0.976(1.5%)	0.973(1.4%)
MOLLI T1	0.978(1.5%)	0.978(1.2%)	0.982(1.1%)

ICC: intraclass correlation coefficient; COV: coefficient of variance.

## Discussion

As previously described, decreased native myocardial T1 is highly prevalent in Fabry disease patients with LVH. We show in addition that in subjects without LVH, reduced myocardial T1 has a 50% prevalence and is associated with echocardiographic parameters of cardiac dysfunction suggesting that a low T1 is detecting early cardiac disease. As a candidate biomarker, native myocardial T1 in Fabry disease is useful both in established and early disease, and with both MOLLI and ShMOLLI approaches.

There are many current clinical challenges in Fabry disease. For example, the diagnosis is often missed and there is uncertainty as when to begin ERT and also how to monitor its effects. CMR derived myocardial T1 mapping has previously been shown to have very high sensitivity and specificity to discriminate Fabry disease patients with LVH [12,13]. Low T1 in Fabry disease is likely a consequence of the progressive sphingolipid storage in the myocardium. We suspect that it may be the specific storage pattern of lipid in Fabry disease i.e. the lamellar bodies with water restricted between layers, reminiscent of the myelin sheath rather than just the presence of fat [13].

We have previously proposed 4 phases of myocardial Fabry disease (with these data adding myocardial dysfunction to phase 2): phase 1: normal; phase 2: low T1, early myocardial dysfunction; phase 3, LVH low T1 and phase 4: ‘pseudonormalisation’ of T1, fibrosis and heart failure. This study shows that low T1 is not quite ubiquitous in LVH positive patients with 4 patients not having a reduced septal T1. Whilst one of these may have been in a progressive disease phase 4, where scarring is causing “pseudonormalisation” of T1, the other 3 were female heterozygotes with early disease or with an apical HCM like LVH pattern and were on ERT. One tantalising explanation is that ERT in early disease may normalise T1. The efficacy of ERT appears to decrease with the progression of the disease and may be unsuccessful when marked fibrosis has been established [6,22]. If storage can be removed before the myocyte and myofibroblast activate and induce hypertrophy and fibrosis, cardiac disease could be avoided. The link of low T1 to early dysfunction may indicate the early stages of such activation. These 50% of LVH negative individuals with a low T1 could therefore be a target for early ERT. The reproducibility data for native T1 has been documented in other diseases [10,20,23,24] and here appears to be a reliable marker in Fabry disease, at least when performed on the same magnet. There are ongoing research programs working on standardization between magnets.

This reproducibility is not found in advanced echocardiographic parameters where increased sensitivity between groups (here lower global longitudinal strain and a worse E/E’ ratio) has not yet been matched with robust inter-subject reproducibility and the ability to guide therapy in an individual [19,25-28]. This provides three potential advantages: first, a potentially clear risk stratification and therapy targeting method; secondly, a way of clinically monitoring therapy; and thirdly, a surrogate endpoint for drug development, dose ranging and clinical trials. An integrated imaging approach including both cardiac magnetic resonance and echocardiographic parameters might be optimal for the management of these patients.

### Limitations

CMR, ECG and echocardiographic findings were not compared to the gold standard of cardiac biopsy, or candidate measures of myocardial storage such as CMR derived spectroscopy. The reproducibility work is single centre, single magnet and therefore further work will be required to assess these variables in scanners from different vendors and at 3 Tesla field strength. A potential limitation of the family of T1 mapping methods used in this paper is the dependence of the T1 values on other physical parameters such as T2 and magnetization transfer (MT), which have been shown to affect apparent T1 values [29].

Furthermore, because of small numbers especially in LVH negative group, separate analysis by gender or prior ERT use were prevented. Future work will need to be larger, multicentre and look at both disease progression and impact of therapy. Lastly, we didn’t measure extracellular volume (ECV) in our population. Because ECV is a ratio, ECV might be more comparable across platforms and sequences. However, we didn’t measure ECV based on previous data from our center, showing ECV didn’t raise in AFD patients.

## Conclusions

Native T1 mapping is highly reproducible in Fabry disease using either ShMOLLI or MOLLI sequences. In Fabry disease LVH negative individuals, low native T1 tracks early myocardial dysfunction and appears to be a promising tool to detect early cardiac involvement in Fabry disease individuals.
